# Association of hypercholesterolemia and cardiac function evaluated by speckle tracking echocardiography in a rabbit model

**DOI:** 10.1186/1476-511X-13-128

**Published:** 2014-08-09

**Authors:** Liyun Liu, Yuming Mu, Wei Han, Chunmei Wang

**Affiliations:** Department of Echocardiography, Center of Medical Ultrasound, First Affiliated Hospital of Xinjiang Medical University, No. 137, Li Yu Shan South Road, Urumqi, 830011 China

**Keywords:** Hypercholesterolemia, Myocardial function, Speckle tracking echocardiography

## Abstract

**Background:**

Although hypercholesterolemia is a major risk factor for coronary artery disease (CAD), only limited data are available regarding its direct effect on myocardial function apart from CAD. The aim of this study was to evaluate LV systolic function using speckle-tracking echocardiography and investigate the relationship between hypercholesterolemia and myocardial function.

**Methods:**

Twenty-eight rabbits were randomly divided into three groups: 8 were fed normal chow for 3 months (group 1) and the remaining 20 were fed an atherogenic diet for 2 (group 2) or 3 months (group 3). Global systolic radial, circumferential and longitudinal peak strain were calculated. Serum total cholesterol (TC), low density lipoprotein cholesterol (LDL-C) and myocardial cholesterol levels were measured.

**Results:**

Global systolic longitudinal strain were both decreased in the group 2 and 3 (*P* < 0.001), whereas radial strain were increased (*P* < 0.001) compared with group 1. Global circumferential strain in the group 3 was significantly reduced (*P* < 0.001). Serum and myocardial cholesterol concentration markedly increased in the group 2 and group 3 (*P* < 0.001). There was a significant inverse correlation between longitudinal strain and serum TC, LDL-C as well as myocardial cholesterol levels (*r* = - 0.723, *r* = - 0.794, *r* = - 0.700, *P* both < 0.001). A significant negative correlation was also noted between circumferential strain and serum TC, LDL-C as well as myocardial cholesterol levels (*r* = - 0.518, *P* = 0.007; *r* = - 0.691, *P* < 0.001; *r* = - 0.659, *P* < 0.001). A significant positive correlation was found between radial strain and serum TC, LDL-C as well as myocardial cholesterol levels (*r* = 0.432, *P* = 0.028; *r* = 0.602, *P* = 0.001; *r* = 0.469, *P* = 0.016).

**Conclusion:**

Although LV morphology and ejection fractions were not different among the three groups, elevated concentration of cholesterol, especially in serum LDL-C, was significantly associated with LV systolic dysfunction. The findings also indicate that reductions in longitudinal was the first appeared, followed by circumferential, and was compensated for by increasing radial strain.

## Background

Although hypercholesterolemia has emerged as a strong risk factor for coronary artery disease (CAD) [1–3], only limited data are available regarding its direct effect on myocardial function apart from CAD [4–6]. The metabolic derangement of hypercholesterolemia can result in abnormalities of cardiac function that are likely independent of effects on the vasculature [5]. While single left ventricular (LV) myocytes isolated from hypercholesterolemic rabbits demonstrated a significant reduction in systolic function without any change in blood pressure or LV morphology [4], few data are available from in vivo investigations.

Speckle tracking echocardiography (STE), a relatively new echocardiographic imaging modalities, offers an objective and quantitative evaluation of global and regional myocardial deformation in longitudinal, radial and circumferential directions [7–9]. A large amount of published data has described that STE could detect subtle changes in LV function at an early subclinical stage [10–13].

The aim of the present study was to elucidate whether dietary hypercholesterolemia alters LV systolic function independently of CAD using STE in rabbits model and investigate their relationship.

## Methods

### Animal model

The experimental protocol was approved by a local ethical committee (First Affiliated Hospital, Xinjiang Medical University, Xinjiang, China). Twenty-eight male New Zealand rabbits (1.9-2.3 kg) were housed in separate cages in an environmentally controlled facility (AAALAC accredited) and were given water ad libitum and received humane care in compliance with institutions guidelines. The rabbits were acclimatized to laboratory conditions for 7 days prior to treatment. Eight rabbits were fed normal chow for 3 months as control (group 1) and the remaining 20 accepted an atherogenic diet for 2 months (group 2) or 3 months (group 3). The atherogenic diet contained 84% standard chow diet, 5% lard, 5% egg yolk powder and 2% cholesterol [13]. Diarrhea, appetite and coat color were observed during the experimental period.

### Echocardiographic imaging

On the day of the study, rabbits fast for approximately 4 h to reduce abdominal distention and to facilitate obtaining the images. Echocardiographic images were acquired after lightly sedated with 10 mg/kg ketamine (Fujian Gutian Pharmaceutical Co., Ltd, China), 1 mg/kg Diazepam (Tianjin Jinyao Amino Acid Co., Ltd, China) and 0.025 mg/kg Atropine (Tianjin Pharmaceutical Group Co., Ltd, China) administered intravenously. The rabbits were placed in prone position without restraint. All images were obtained using a commercial ultrasound machine (Vivid 7 Dimension; GE Vingmed Ultrasound AS, Horten, Norway) with an M5S probe. M- mode images of parasternal long-axis view, B-mode images of apical three-chamber, four-chamber, and two-chamber views, short-axis views at the level of the mitral valve, papillary muscles, and apex were obtained and digitally stored in cine-loop format for off line analysis [14]. LV end-diastolic diameter (LVEDd), LV end-systolic diameter (LVEDs), septal and LV posterior wall thickness, and left atrial anteroposterior diameter (LAD) were measured from standard planes. LV ejection fraction (EF) was calculated with the Teicholz formula [15].

### Strain analysis

**Figure 1:**
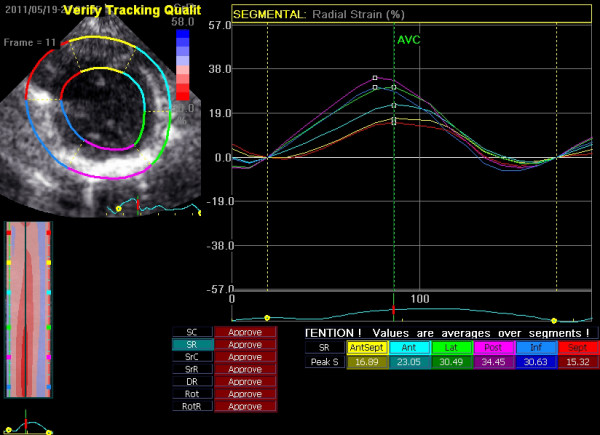
**Systolic radial stain of the six myocardial segments.**

Two-dimensional B-mode images were captured with a frame rate of 50–80 fps and five beats were recorded for analysis. Blinded offline analyses of the short-axis views and apical long-axis views were performed using EchoPAC PC version 6.1.1 (GE Vingmed Ultrasound AS, Horten*,* Norway). After selecting the best-quality image of the cardiac cycle, the LV endocardial border was manually traced at the end-systolic frame, from which a speckle-tracking region of interest was automatically selected to approximate the myocardium between the endocardium and epicardium [16]. The workstation then computed and generated strain curves. The software automatically divided the sectional image into six segments according to the statement of the Cardiac Imaging Committee of the Council on Clinical Cardiology of the American Heart Association [17] (Figure 1). Strain curves of three consecutive cardiac cycles and values were imported for further analysis. To determine global longitudinal, circumferential and radial strain, the strain values of the 18 segments were averaged for the apical views or the short-axis views.

### Blood pressure measurement

After echocardiographic imaging, blood pressure measurements were made from the central ear artery (CEA) of rabbits using a 20G vascular catheter (Johnson and Johnson, Belgium). The arterial catheter was connected to a pressure transducer (MLT0699, AD Instruments, Pty Ltd, Australia) and an analog-to-digital converter (PowerLab, ML866, AD Instruments, Pty Ltd, Australia). Arterial systolic and diastolic pressure were recorded.

### Cholesterol analysis

Peripheral blood was collected from ear veins with a 25-gauge needle and syringe at baseline, 2 months and 3 months. Total cholesterol (TC) and low density lipoprotein cholesterol (LDL-C) were measured with an automated clinical chemistry analyzer (AU680, Beckman Instruments, USA). All rabbits were euthanized at correspnding time and the hearts removed. A segment of myocardial tissue (10 × 3 mm) from the LV free wall was excised. Cholesterol levels were measured in the tissue segment using a cholesterol Kit (EnzyChrom Cholesterol Assay Kit, BioAssay Systems, Hayward, CA).

### Statistical analysis

SPSS 16.0 (SPSS inc., Chicago, Illinois, USA) was used for statistical analysis. The data were tested for normality and homogeneity of variance. Data are expressed as mean ± standard deviation (SD). One way ANOVA was used to compare the echocardiographic parameters, strain parameters, myocardial and serum cholesterol levels for all three groups. Pearson correlation analysis was done between STE variables and cholesterol levels. For all analyses, a *P* value < 0.05 was considered significant. Interobserver and intraobserver variability for strain measurements were examined using both Pearson’s bivariate two-tailed correlations and Bland-Altman analysis from 10 randomly selected rabbits.

## Results

### Animal

Of the 28 experimental rabbits, 1 died in the group 3 due to diarrhea. Heart rates and blood pressure of three groups were similar (*P* > 0.05, Table 1).

**Table 1:** **General characteristics of the study animals**

Parameters	Group 1 n = 8	Group 2 n = 10	Group 3 n = 9	***F***-value	***P***-value
HR (beats/min)	183.50 ± 9.49	168.30 ± 9.32	187.89 ± 11.02	2.21	0.13
SBP (mmHg)	109.88 ± 11.31	110.50 ± 8.37	111.22 ± 10.33	0.12	0.88
DBP (mmHg)	78.25 ± 8.05	77.50 ± 10.84	82.67 ± 10.06	0.73	0.49

HR, heart rate; SBP, systolic blood pressure; DBP, diastolic blood pressure.

### Conventional echocardiography

Echocardiographic measurements of the different groups were shown in Table 2. There were no significant differences in LVEF, LVEDd, LVEDs, IVS, PW and LAD among three groups (*P* > 0.05).

**Table 2:** **Comparison of echocardiographic parameters**

Parameters	Group1 n = 8	Group2 n = 10	Group3 n = 9	***F***-value	***P***-value
LVEDd (mm)	13.14 ± 1.43	13.51 ± 1.69	13.33 ± 1.58	0.12	0.89
LVEDs (mm)	8.62 ± 0.86	9.00 ± 1.02	8.81 ± 0.92	0.37	0.70
IVS (mm)	2.12 ± 0.31	1.88 ± 0.36	2.08 ± 0.37	1.28	0.30
PW (mm)	2.04 ± 0.22	2.01 ± 0.20	2.23 ± 0.38	1.63	0.22
LVEF (%)	67.38 ± 4.63	65.97 ± 4.61	66.93 ± 3.73	0.25	0.78
LAD (mm)	8.84 ± 1.15	9.94 ± 1.23	9.42 ± 0.99	0.66	0.53

LVEDd, left-ventricular end-diastolic diameter; LVEDs, left-ventricular end-systolic diameter; EF, ejection fraction; LAD, left-atrial diameter; IVS, ventricular septal end-diastolic thickness; PW, posterior wall end-diastolic thickness.

### Strain measurements

**Figure 2:**
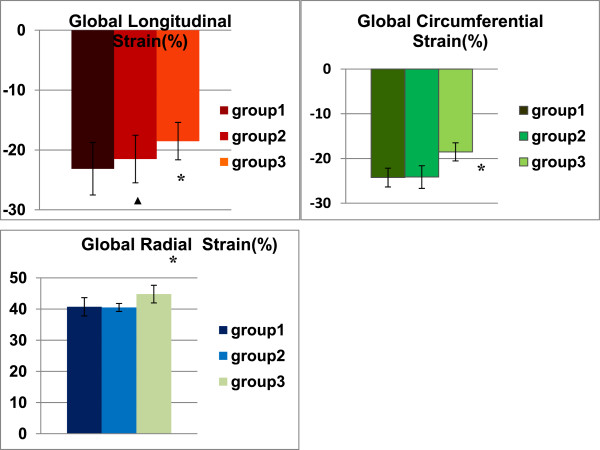
Global peak systolic longitudinal, circumferential and radial strain in the three groups. *p < 0.001 for group 3 vs. group 2 and group 1. ^▲^p < 0.05 for group 2 vs group 1.

From a total of 972 analyzed segments, 28 segments were excluded owing to suboptimal myocardial tracking and poor image quality. Regional longitudinal (Table 3) and circumferential (Table 4) strain of LV were significantly reduced in group 3 compared with group 1 and 2. But regional radial strain of LV were significantly increased in group 3 (Table 5).Global longitudinal myocardial deformation of the LV was significantly impaired both in group 2 and 3, while radial deformation was increased in group 3 compared with group 1 and 2. In addition, global circumferential strain was also reduced in group 3 compared with group 1 and 2 (Figure 2).

**Table 3:** **Peak systolic longitudinal strain values**

Longitudinal strain (%)	Group 1 n = 8	Group 2 n = 10	Group 3 n = 9	***F***-value	***P***-value
A4C septum					
Basal segment	-21.76 ± 3.28	-21.97 ± 4.08	-18.87 ± 2.15	2.240	0.129
Mid segment	-23.40 ± 4.38	-21.89 ± 3.47	-18.91 ± 2.20*	3.493	0.047
Apical segment	-24.54 ± 5.57	-22.08 ± 4.32	-19.41 ± 3.06*	2.685	0.090
A4C lateral wall					
Basal segment	-23.64 ± 4.44	-21.96 ± 3.31	-18.30 ± 1.69*^▲^	5.382	0.019
Mid segment	-20.88 ± 3.74	-20.98 ± 3.20	-18.32 ± 2.01	1.945	0.166
Apical segment	-22.94 ± 3.36	-21.90 ± 3.48	-19.28 ± 4.21	2.115	0.143
A3C posterior wall					
Basal segment	-22.74 ± 3.21	-20.89 ± 2.38	-18.87 ± 3.35*	3.425	0.050
Mid segment	-21.71 ± 2.68	-22.05 ± 2.65	-18.57 ± 3.13*^▲^	3.924	0.034
Apical segment	-23.77 ± 2.78	-21.15 ± 3.47	-21.60 ± 4.27	1.327	0.285
A3C anterior septum					
Basal segment	-21.31 ± 3.34	-20.92 ± 3.54	-19.41 ± 2.79	0.764	0.477
Mid segment	-21.96 ± 2.95	-21.09 ± 3.70	-19.62 ± 3.68	0.837	0.446
Apical segment	-22.28 ± 2.24	-22.59 ± 2.87	-18.86 ± 3.55*^▲^	4.197	0.028
A2C inferior wall					
Basal segment	-25.78 ± 4.34	-20.90 ± 2.74*	-19.36 ± 3.19*	7.749	0.003
Mid segment	-23.37 ± 4.53	-20.02 ± 2.59	-19.08 ± 3.61*	3.205	0.059
Apical segment	-24.54 ± 4.51	-21.23 ± 2.34	-21.57 ± 4.23	2.027	0.155
A2C anterior wall					
Basal segment	-23.64 ± 4.28	-21.75 ± 2.34	-18.96 ± 3.24*	4.066	0.031
Mid segment	-23.17 ± 3.39	-23.37 ± 2.68	-17.82 ± 3.00*^▲^	9.140	0.001
Apical segment	-24.28 ± 5.70	-23.59 ± 3.90	-21.10 ± 4.14	1.075	0.358

Data are expressed as mean ± SD. A4C, Apical four-chamber; A3C, apical three-chamber; A2C, apical two-chamber. *p < 0.05 for group 3 and group 2 vs. group 1, ^▲^ *p* < 0.05 for group 3 vs. group 2.

**Table 4:** **Peak systolic circumferental strain values**

Circumferental strain (%)	Group 1 n = 8	Group 2 n = 10	Group 3 n = 9	***F***-value	***P***-value
Mitral valve level					
Anteroseptal wall	-30.18 ± 5.52	-30.33 ± 5.91	-23.45 ± 6.55*^▲^	3.571	0.045
Anterior wall	-23.76 ± 4.86	-23.22 ± 4.42	-20.09 ± 2.33	1.939	0.167
Lateral wall	-20.63 ± 5.89	-19.75 ± 3.35	-19.10 ± 3.19	0.263	0.771
Posterior wall	-21.40 ± 4.46	-20.85 ± 4.58	-18.65 ± 3.25	0.869	0.433
Inferior wall	-22.58 ± 4.22	-20.89 ± 4.59	-17.34 ± 3.73*	3.215	0.059
Septal wall	-27.99 ± 6.30	-27.83 ± 6.71	-21.75 ± 4.45*^▲^	2.937	0.073
Papillary level					
Anteroseptal wall	-28.22 ± 7.03	-29.73 ± 6.00	-24.80 ± 4.06	1.619	0.22
Anterior wall	-20.58 ± 3.78	-22.99 ± 4.44	-19.76 ± 2.89	1.776	0.192
Lateral wall	-18.99 ± 3.19	-20.18 ± 3.17	-18.31 ± 2.04	0.982	0.39
Posterior wall	-19.32 ± 3.52	-19.73 ± 3.31	-19.45 ± 1.06	0.047	0.954
Inferior wall	-21.01 ± 3.60	-20.59 ± 2.33	-17.19 ± 1.89*^▲^	5.047	0.015
Septal wall	-27.98 ± 7.59	-28.21 ± 5.53	-20.66 ± 3.03*^▲^	4.717	0.019
Apical level					
Anteroseptal wall	-30.24 ± 6.67	-28.71 ± 6.29	-21.53 ± 3.78*^▲^	5.284	0.013
Anterior wall	-25.66 ± 5.46	-23.68 ± 5.03	-19.68 ± 2.60*	3.539	0.046
Lateral wall	-24.88 ± 4.33	-22.72 ± 6.18	-19.48 ± 3.25*	2.192	0.134
Posterior wall	-25.33 ± 5.92	-23.65 ± 3.84	-19.49 ± 3.06*	3.782	0.038
Inferior wall	-25.81 ± 5.45	-23.46 ± 5.93	-18.66 ± 2.86*	4.228	0.027
Septal wall	-26.64 ± 6.78	-27.42 ± 6.34	-24.85 ± 5.08	0.553	0.582

Data are expressed as mean ± SD. *p < 0.05 for group 3 and group 2 vs. group 1, ^▲^p < 0.05 for group 3 vs. group 2.

**Table 5:** **Peak systolic radial strain values**

Radial strain (%)	Group 1 n = 8	Group 2 n = 10	Group 3 n = 9	***F***-value	***P***-value
Mitral valve level					
Anteroseptal wall	36.84 ± 6.98	38.88 ± 6.16	45.48 ± 6.85* ^▲^	3.756	0.039
Anterior wall	37.92 ± 7.15	40.48 ± 8.06	44.77 ± 9.82	1.368	0.275
Lateral wall	41.65 ± 7.78	42.02 ± 9.12	46.48 ± 9.37	0.765	0.477
Posterior wall	39.65 ± 5.41	44.31 ± 7.06	49.07 ± 6.21*	4.426	0.024
Inferior wall	40.92 ± 5.31	42.54 ± 7.64	50.13 ± 7.02* ^▲^	4.231	0.027
Septal wall	38.09 ± 7.21	40.84 ± 7.21	47.40 ± 5.41*	4.098	0.03
Papillary level					
Anteroseptal wall	37.41 ± 5.01	38.11 ± 5.38	44.81 ± 10.12*	2.726	0.087
Anterior wall	37.06 ± 7.45	37.83 ± 6.56	39.28 ± 7.31	0.205	0.816
Lateral wall	42.32 ± 7.81	39.45 ± 7.27	42.05 ± 9.37	0.351	0.708
Posterior wall	42.54 ± 7.14	44.24 ± 3.81	46.22 ± 11.70	0.432	0.654
Inferior wall	43.18 ± 8.54	42.40 ± 4.87	45.67 ± 6.38	0.574	0.571
Septal wall	39.85 ± 8.45	41.14 ± 6.87	48.49 ± 6.96* ^▲^	3.23	0.058
Apical level					
Anteroseptal wall	39.10 ± 6.70	38.63 ± 5.90	43.96 ± 6.63	1.802	0.187
Anterior wall	37.29 ± 6.44	38.84 ± 5.84	44.71 ± 4.59* ^▲^	3.88	0.035
Lateral wall	43.85 ± 8.84	40.85 ± 3.97	47.66 ± 9.67* ^▲^	3.159	0.061
Posterior wall	41.17 ± 7.37	41.74 ± 6.88	44.07 ± 12.15	0.241	0.788
Inferior wall	44.52 ± 6.66	42.14 ± 8.85	44.90 ± 7.49	0.336	0.718
Septal wall	42.44 ± 6.06	38.34 ± 5.84	46.96 ± 10.41^▲^	2.88	0.076

Data are expressed as mean ± SD. *p < 0.05 for group 3 and group 2 vs. group 1, ^▲^p < 0.05 for group 3 vs. group 2.

### Serum and tissue cholesterol profiles

The serum cholesterol profiles of the three groups after experiment were shown in Table 6. There was a statistically significant increase in serum TC, LDL-C and tissue cholesterol levels in animals fed with cholesterol enriched diet compared with the control group (p <0.05). Morerove, the concentration of cholesterol increased with feeding duration (*p* <0.05).

**Table 6:** **Serum and tissue cholesterol profiles**

Parameters	Group 1 n = 8	Group 2 n = 10	Group 3 n = 9	***F***-value	***P***-value
Serum TC (mmol/L)	2.07 ± 0.60	24.15 ± 5.36*	34.74 ± 10.40* ^▲^	191.27	0.000
Serum LDL-C (mmol/L)	1.13 ± 0.54	10.73 ± 3.32*	31.62 ± 3.68* ^▲^	229.60	0.000
Tissue cholesterol(μmol/g)	0.86 ± 0.29	2.22 ± 0.62*	4.92 ± 1.63* ^▲^	34.68	0.000

Data are expressed as mean ± SD. TC, total cholesterol; LDL-C, low density lipoprotein cholesterol.

*p < 0.05 for group 3 and group 2 vs. group 1, ^▲^p < 0.05 for group 3 vs. group 2.

### Correlation between strain parameters and cholesterol levels

The correlation between strain parameters and cholesterol were shown in Table 7. There was significant inverse correlation between global longitudinal strain and serum TC, LDL-C as well as myocardial cholesterol levels. (r = - 0.723, P < 0.001; r = - 0.794, P < 0.001; r = - 0.70, P < 0.001). A significant negative correlation was also noted between global circumferential strain and serum TC, LDL-C as well as myocardial cholesterol levels. (r = - 0.518, P = 0 .007; r = - 0.691, P < 0.001; r = - 0.659, P < 0.001). A significant positive correlation was found between radial strain and serum TC, LDL-C as well as myocardial cholesterol levels. (r = 0.432, P = 0.028; r = 0.602, P = 0.001; r = 0.469, P = 0.016).

**Table 7:** **Correlation between strain paramters and cholesterol**

Strain parameters	Serum TC		Serum LDL -C		Tissue cholesterol	
	***r***	***p***	***r***	***p***	***r***	***p***
GLS	-0.723	0.000	-0.794	0.000	-0.700	0.000
GRS	0.432	0.028	0.602	0.001	0.469	0.016
GCS	-0.518	0.007	-0.691	0.000	-0.659	0.000

GLS, global longitudinal strain; GRS, global radial strain; GCS, global circumferential strain; TC, total cholesterol; LDL-C, low density lipoprotein cholesterol.

### Reproducibility

**Figure 3:**
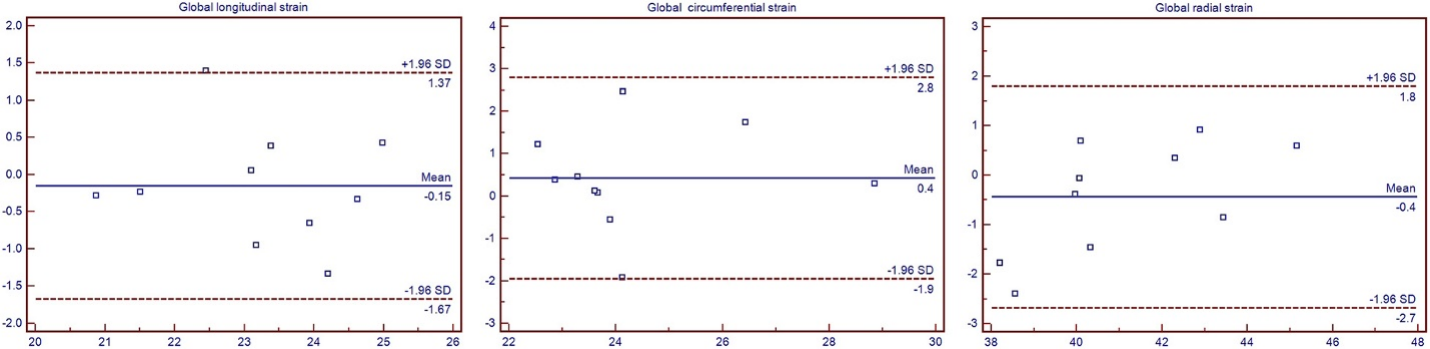
**Inter-observer variability for global longitudinal (left), circumferential (middle) and radial strain (right).**

The results showed very good intra-observer variability for longitudinal, circumferential and radial strain rate (r = 0.817, P = 0.004; r = 0.798, P = 0.006; r = 0.868, P = 0.001). The Bland-Altman plots demonstrated acceptable inter-observer variability for all strain parameters (Figure 3).

## Discussion

To the best of our knowledge, the present study is the first to comprehensively compare strain parameters-derived STI with the levels of serum and myocardial cholesterol in diet-induced expeimental hypercholesterolemia. Although previous experimental studies have shown that diet-induced hypercholesterolemia resulted in contractile reduction of single ventricular myocyte without any change in pressure or LV morphology [4], few data are available from in vivo investigations. The present study displayed the application of STE as a noninvasive imaging technique to elucidate the direct effect of hypercholesterolemia on LV myocardial deformation in a rabbit model.

In our study, there were no significant differences in LV morphology, EF and blood pressure among groups, whereas LV strain was found to be reduced in the hypercholesterolemic rabbits. Previous studies failed to show abnormalities using EF, which may be due to EF reflects the whole LV systolic function, under the influence of pre and afterload [18]. With the application of advanced techniques, such as strain, strain rate, incipient systolic dysfunction has been detected in subclinical diseases [19]. Moreover, our analysis indicates that longitudinal dysfunction are the first appeared, followed by circumferential, which suggest the importance of longitudinal strain in the assessment of LV systolic dysfunction in subclinical stage.

Whether similar changes occur in humans with hypercholesterolemia can not confirm from our study. However, a recent human study demontrated longitudinal and circumferential deformations were both impaired in the children with heterozygous familiar hypercholesterolemia [20]. Thus, we believe that the abnormalities we found in rabbit models with hypercholesterolemia indicate an early sign of hypercholesterolemia-induced myocardial dysfunction, in agreement with the in vitro expriments [4].

Interestingly, our study demonstrates that the increased radial deformation make up for impaired longitudinal and circumferential strain in rabbit hypercholesterolemic models to maintain LVEF. This finding is consistent with prior reports in children with heterozygous familial hypercholesterolemia and other preclinical diseases [14, 20, 21]. The potential mechanism by which hypercholesterolemia causes the increase in radial deformation remains unclear. A possible explanation could be the realignments of myocardial fiber orientation in the outer half of the myocardium may contribute to “transmural compensation” by less impaired epicardial fibers [22].

In the present study, a significant negative correlation were found between global longitudinal strain and serum cholesterol level as well as myocardial cholesterol levels. These results indicate that the cholesterol accumulated in the myocardium may be responsible for a reduction in myocardial strain. Similar to our study, Wang et al. [23] reported a positive correlation between serum HDL levels and LVEF in human subjects with serum hypercholesterolemia even in the absence of angiographic evidence of CAD.

The precise mechanism responsible for the association between cholesterol level and impaired myocardial deformation cannot be determined from our study. However, several mechanisms have been proposed to explain LV dysfunction induced by hypercholesterolemia: (1) increased cardiac oxidative stress [24], (2) alteration of the myocardial energy metabolism [22], (3) changes in myosin heavy-chain isoform expression patterns [4], (4) down-regulation and redistribution of connexin-43 expression in myocardium [25], and (5) impaired activation of myocardial adenosine triphosphate-sensitive potassium channels [19]. These mechanisms may represent the basis for a “hypercholesterolemic cardiomyopathy [26].

### Study limitations

As a limitations of our study, administration of ketamine- Diazepam - Atropine combinations induces mild bradycardia, which slightly alters cardiac function. In addition, LV diastolic function, rotation and torsion mechanics are potentially very important features for the comprehensive understanding of myocardial tissue damage; therefore, lack of measurement of diastolic function, rotation and torsion was another limitation of the present study.

## Conclusion

Hypercholesterolemia was significantly associated with LV myocardial functional alterations apart from CAD. The findings also indicate that decreases in longitudinal was the first appeared, followed by circumferential, and was compensated for by increasing radial strain. Thus, the application of STE may provide noninvasive functional insight into disease progression or recovery in reponse to therapeutic intervention.

## Abbreviations

CAD: Coronary artery disease
STE: Speckle-tracking echocardiography
LV: Left ventricular
TC: Total cholesterol
LDL-C: Low density lipoprotein cholesterol
LVEDd: LV end-diastolic diameter
LVEDs: LV end-systolic diameter
CEA: Central ear artery.
